# Triple-network model–based graph theory analysis of the effectiveness of low-dose ketamine in patients with treatment-resistant depression: two resting-state functional MRI clinical trials

**DOI:** 10.1192/bjp.2025.14

**Published:** 2025-11

**Authors:** Wei-Chen Lin, Li-Kai Cheng, Tung-Ping Su, Li-Fen Chen, Pei-Chi Tu, Cheng-Ta Li, Ya-Mei Bai, Shih-Jen Tsai, Mu-Hong Chen

**Affiliations:** Department of Psychiatry, Taipei Veterans General Hospital, Taipei, Taiwan; Division of Psychiatry, School of Medicine, College of Medicine, National Yang Ming Chiao Tung University, Taipei, Taiwan; Department of Medical Research, Taipei Veterans General Hospital, Taipei, Taiwan; Institute of Brain Science, National Yang Ming Chiao Tung University, Taipei, Taiwan; Brain Research Centre, National Yang Ming Chiao Tung University, Taipei, Taiwan; Institute of Philosophy of Mind and Cognition, National Yang Ming Chiao Tung University, Taipei, Taiwan; Department of Psychiatry, General Cheng Hsin Hospital, Taipei, Taiwan

**Keywords:** Triple-network model, ketamine, treatment-resistant depression, graph theory, resting-state functional connectivity

## Abstract

**Background:**

Evidence suggests the crucial role of dysfunctional default mode (DMN), salience and frontoparietal (FPN) networks, collectively termed the triple network model, in the pathophysiology of treatment-resistant depression (TRD).

**Aims:**

Using the graph theory- and seed-based functional connectivity analyses, we attempted to elucidate the role of low-dose ketamine in the triple networks, namely the DMN, salience and FPN.

**Method:**

Resting-state functional connectivity magnetic resonance imaging (rs–fcMRI) data derived from two previous clinical trials of a single, low-dose ketamine infusion were analysed. In clinical trial 1 (Trial 1), patients with TRD were randomised to either a ketamine or normal saline group, while in clinical trial 2 (Trial 2) those patients with TRD and pronounced suicidal symptoms received a single infusion of either 0.05 mg/kg ketamine or 0.045 mg/kg midazolam. All participants underwent rs–fcMRI pre and post infusion at Day 3. Both graph theory- and seed-based functional connectivity analyses were performed independently.

**Results:**

Trial 1 demonstrated significant group-by-time effects on the degree centrality and cluster coefficient in the right posterior cingulate cortex (PCC) cortex ventral 23a and b (DMN) and the cluster coefficient in the right supramarginal gyrus perisylvian language (salience). Trial 2 found a significant group-by-time effect on the characteristic path length in the left PCC 7Am (DMN). In addition, both ketamine and normal saline infusions exerted a time effect on the cluster coefficient in the right dorsolateral prefrontal cortex a9-46v (FPN) in Trial 1.

**Conclusions:**

These findings may support the utility of the triple-network model in elucidating ketamine’s antidepressant effect. Alterations in DMN, salience and FPN function may underlie this effect.

Over the past two decades, a substantial body of evidence has substantiated the promising antidepressant efficacy of low-dose ketamine in the treatment of treatment-resistant depression (TRD) and associated suicidal symptoms.^1–3^ Ketamine’s antagonistic interaction with N-methyl-D-aspartate (NMDA) receptors triggers molecular processes in the mammalian target of the rapamycin pathway, further promoting brain-derived neurotrophic factor release and synaptogenesis.^1–3^ However, the neurobiological mechanisms underlying the rapid antidepressant actions of low-dose ketamine remain unclear.

Dysfunction in three principal brain networks – namely dysfunctional default mode (DMN), salience and frontoparietal (FPN), collectively termed the triple-network model – has been implicated in the pathophysiology of major depressive disorder and TRD.^4,5^ Wang et al reported decreased functional connectivity between the right habenula and bilateral angular gyrus (DMN hubs) at post-infusion in treatment responders, compared with non-responders, to six infusions of 0.05 mg/kg ketamine in patients with moderate-to-severe depression or bipolar disorder.^6^ Alexander et al emphasised the pivotal role of the anterior cingulate cortex (ACC), a salience hub, in the neuromechanism underlying ketamine’s antidepressant effect, and reported rapid and sustained modulations in ACC activity following low-dose ketamine administration.^7^ Our previous [^18^F]Fluorodeoxyglucose positron emission tomography (PET) study revealed a significant correlation between increased ACC glucose metabolism and reduced depressive symptoms, as measured using the 17-item Hamilton Rating Scale for Depression.^8^ Additionally, a rapid increase in prefrontal cortex (PFC) glucose metabolism was noted following low-dose ketamine infusion among patients with TRD.^8^ An open-label, resting-state functional magnetic resonance imaging (rs–fMRI) study of 16 patients with depression who were given a single infusion of 0.05 mg/kg ketamine demonstrated that a more pronounced effect of ketamine on cognitive symptoms was associated with lower DMN deactivation and higher dorsolateral prefrontal cortex (DLPFC, a FPN hub) activation.^9^ However, the DMN, salience and FPN never act singly but only in concert as a well-regulated entity.^4^ Thus, these three principal networks must be treated as an interconnected entity if any insight into the effects of ketamine is to be gained. In addition, increasing evidence has shown that traditional network hubs are typically highly connected, but changes within non-hub regions may reflect subtle but important network reconfiguration.^10,11^ In order to elucidate more broadly the network reconfiguration among DMN, salience and PFN, the HCPex atlas was used in our study to define a much larger selection of regions of interest (ROIs).^10,12^

The present study investigated the triple-network model hypothesis regarding ketamine’s antidepressant effects on patients with TRD through a graph theory analysis of rs–fMRI data. Specifically, we calculated graph theory-driven metrics: degree centrality, clustering coefficient, characteristic path length and transitivity. Based on previous findings on the effect of low-dose ketamine on the DMN, salience and FPN individually, the present study attempted to clarify the effect of ketamine on the interaction among those three networks according to triple-network models. We hypothesised that low-dose ketamine infusion would change the graph theory metrics of the DMN, salience and FPN relative to placebo among patients with TRD.

## Method

### Participants

Two double-blind, placebo-controlled, randomised clinical trials (Trial 1^2^ and Trial 2^3^) of low-dose ketamine infusion in patients with major depressive disorder were conducted between 2013 and 2021 (Supplementary Figs 1 and 2 available at https://doi.org/10.1192/bjp.2025.14). According to the *Diagnostic and Statistical Manual of Mental Disorders*, fifth edition,^13^ all participants had major depressive disorder and did not respond adequately to at least two different antidepressants at the adequate dosage and for the treatment duration. In Trial 1 we enrolled patients with TRD, while in Trial 2 we enrolled patients with TRD and strong suicidal symptoms. Strong suicidal symptoms were defined as a score of ≥4 on the Montgomery–Åsberg Depression Rating Scale (MADRS) item 10, in agreement with Murrough et al.^14^ Exclusion criteria included major medical or neurological diseases, or a history of alcohol or substance use disorders. In Trial 1, patients were randomly assigned to receive a single infusion of either 0.5 or 0.2 mg/kg ketamine or normal saline (placebo). In Trial 2, patients were randomly administered a single infusion of either 0.5 mg/kg ketamine or 0.045 mg/kg midazolam (placebo). Depressive symptoms were assessed using MADRS immediately before infusion (baseline) and on Day 3 post-infusion. In Trial 1, 0.2 and 0.5 mg/kg ketamine infusions were combined into a single ketamine infusion group in neuroimaging analyses, because of the similar clinical outcomes between groups (symptom reduction rate: total MADRS, 37.1% in the 0.2 mg/kg group *v*. 32.6% in the 0.5 mg/kg group, *P* = 0.652). This ketamine group was then compared with the normal saline (placebo) group. We excluded the imaging data of three participants in Trial 1 and four in Trial 2, due to poor image quality; these exclusions were necessary to ensure the reliability and validity of the neuroimaging analysis. This study was performed in accordance with the Declaration of Helsinki and was approved by the Taipei Veterans General Hospital Institutional Review Board (Institutional Review Board numbers 2012-04-037B and 2016-08-001C), and all participants provided written informed consent. The studies were registered with the UMIN Clinical Trials Registry (numbers UMIN000016985 and UMIN000033916).

### Image acquisition

The magnetic resonance images in both Trial 1 and Trial 2 were acquired at Taipei Veterans General Hospital. In Trial 1, images were obtained using a GE 3-Tesla Discovery MR750 scanner (GE Healthcare Life Sciences, Little Chalfont, UK) with a quadrature head coil. Anatomical whole-brain, T1-weighted images were acquired using a magnetisation-prepared, rapid-acquisition, gradient-echo, three-dimensional sequence with the following parameters: repetition time 12.2 ms, echo time 5.2 ms, 168 axial slices, flip angle 12°, field of view (FOV) 256 × 256 mm^2^, matrix size 256 × 256, slice thickness 1 mm. Resting-state functional magnetic resonance images were obtained using a T2*-weighted, gradient-echo approach, echo-planar sequence with the following parameters: repetition time 2500 ms, echo time 30 ms, 43 axial slices, flip angle 90°, voxel size 3.5 × 3.5 × 3.5 mm^3^. In Trial 2, MRI images were acquired using a GE 3-Tesla PET/magnetic resonance scanner (GE Healthcare Life Sciences, Little Chalfont, UK) with a quadrature head coil. Anatomical T1-weighted images were acquired using a similar magnetisation-prepared, rapid-acquisition, gradient-echo sequence with the following parameters: repetition time 6.9 ms, echo time 2.5 ms, 188 axial slices, flip angle 12°, FOV 256 × 256 mm^2^, matrix size 256 × 256, slice thickness 1 mm. Resting-state functional magnetic resonance images were obtained using a T2*-weighted, gradient-echo approach, echo-planar sequence with the following parameters: repetition time 2500 ms, echo time 30 ms, 45 axial slices, flip angle 90°, voxel size 3.5 × 3.5 × 3.6 mm^3^. In both trials, each subject underwent the acquisition of 200 MRI volumes while maintaining closed eyes, a state of mental neutrality and refraining from any movement or falling asleep. Participants were given clear pre-study instructions emphasising these requirements, and trained staff monitored them throughout the session to provide gentle reminders as needed.

### Image data processing

The preprocessing of functional and anatomical data was conducted through a comprehensive pipeline using the CONN toolbox. The initial step involved realignment using the SPM12 (Statistical Parametric Mapping, https://www.fil.ion.ucl.ac.uk/spm/) realign and unwarp procedure. All scans were co-registered to a reference image (the first scan of the first session) using a least-squares approach and a six-parameter (rigid-body) transformation. Subsequently, b-spline interpolation was used for resampling to correct for motion. Temporal misalignment between different slices of the functional data, which were acquired in an interleaved, bottom-up order, was rectified using the SPM slice-timing correction procedure. This involved sinc temporal interpolation to resample each slice blood-oxygen-level dependent (BOLD) time series to a common mid-acquisition time. Outlier scans were identified using Artifact detection tools as acquisitions with framewise displacement >0.9 mm, or as global BOLD signal changes of five or more standard deviations. A reference BOLD image was computed for each subject by averaging all scans, excluding outlier images. Functional and anatomical data were then normalised into standard Montral Neurological Institute space, segmented into grey matter, white matter and cerebrospinal fluid (CSF) tissue classes and resampled to 2 mm isotropic voxels. This was achieved using a direct normalisation procedure incorporating the SPM unified segmentation and normalisation algorithm with the default IXI-549 tissue probability map template. Finally, functional data were smoothed using spatial convolution with a Gaussian kernel of 8 mm full-width, half-maximum. This comprehensive preprocessing pipeline ensured that the data were optimally prepared for subsequent analysis. Moreover, a standard denoising pipeline was applied to the functional data. This involved regressing out potential confounding effects represented by motion parameters (frame displacement) and their first-order derivatives, outlier scans, session effects and first-order derivatives, white matter and CSF time series, and linear trends within each functional run. Subsequently, bandpass frequency filtering of the BOLD time series was conducted, limiting the frequency range between 0.008 and 0.090 Hz.

### Measurement of resting-state network characteristics

ROI-to-ROI connectivity (RRC) matrices were computed to characterise intrinsic functional connectivity between each pair of regions among 39 ROIs. These ROIs were selected from three well-established resting-state networks: DMN (medial prefrontal cortex (MPFC), bilateral lateral parietal cortex and PCC), salience (ACC, bilateral anterior insula, bilateral rostral prefrontal cortex (RPFC) and bilateral SMG) and FPN (bilateral DLPFC and bilateral posterior parietal cortex (PPC)). All resting-state networks were anatomically defined using the CONN functional connectivity toolbox based on *a priori* templates. To ensure accurate spatial representation we utilised the HCPex atlas,^10^ which offers detailed parcellation of the human brain based on resting-state functional connectivity and structural imaging. We verified the spatial overlap between our predefined resting networks and the HCPex atlas, ultimately selecting 39 ROIs from HCPex for further investigation of resting-state network topology. RRC matrices were calculated using Pearson’s correlation coefficients between the mean time series of each ROI pair, followed by Fisher’s *r*-to-*z* transformation to improve normality. The RRC matrices of each subject then further processed through the GRETNA toolbox for the topological properties of the resting network. The degree of an individual node (indicating the importance of that node in the network) represents the number of edges connected to other nodes in this network. Resting-state connectivity was evaluated by computing degree centrality, clustering coefficient, characteristic path length, transitivity and both inter- and intra-network functional connectivity. The degree centrality of node *i*, DC_*i*_, was defined as the summation of all neighbouring edge weights, as follows: DC_*i*_ = 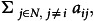 where *a_ij_* is the connection status between node *i* and node *j* and *N* is the set of all nodes in the network. The clustering coefficient of node *i*, CC_*i*_, is equivalent to the fraction of node *i*’s neighbours that are also neighbours of each other. CC_*i*_ is defined as 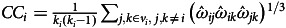 , where 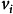 denotes the neighbourhood of node *i*; 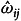 is weight 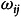 between nodes *i* and *j* scaled by the largest weight in the network; 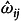 = 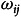 /max( 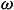 ); and 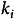 denotes the number of edges connected to node *i*. Characteristic path length is defined as the average number of steps along the shortest paths for all possible pairs of nodes in the network. It quantifies the efficiency of information transfer within the network and is calculated using: 
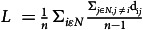
 , where *d* is the shortest distance between nodes *i* and *j* within network *N*, and *n* is the total number of nodes in the network. A shorter characteristic path length indicates a more efficient network structure. Transitivity measures the likelihood that two neighbours of a node are also connected to each other, reflecting local interconnectedness within the network. These metrics collectively provide a comprehensive understanding of resting-state connectivity patterns and their implications for brain function.^15^ In addition to these metrics, intra-network functional connectivity refers to the correlation of activity among regions within the same brain network, calculated by summing functional connectivity values between all pairs of regions within a resting-state network. This metric provides insights into how well these regions synchronise their activity during resting states. Conversely, inter-network functional connectivity assesses connectivity between different brain networks. To calculate inter-network functional connectivity, one typically computes the average functional connectivity between pairs of regions across distinct networks. This analysis helps to elucidate how the activities of various networks interact and are coordinated, which is crucial for understanding brain function and dysfunction.^15^

### Statistical analysis

Statistical analyses were performed utilising SPSS 25 version 25 for Windows (IBM Corp., Armonk, NY, USA) and MATLAB version 9 for Windows (Mathworks, Natick, MA, USA). Two-sample t-tests and Fisher’s chi-square tests were used to analyse continuous and nominal variables, respectively, to evaluate differences between the two infusion groups (ketamine and placebo) concerning demographic and clinical data. Distinctions in network metrics were evaluated using repeated-measures analysis of covariance (ANCOVA), with adjustments for sex and age as covariates (*P* < 0.05), with the infusion group (ketamine *v*. placebo) as a between-patient factor and time (baseline and post-infusion) as a within-patient factor. Post hoc analyses were conducted following significant ANCOVA results to determine specific differences. False discovery rate correction was applied for multiple comparisons to control for Type I error. Paired-sample t-tests were utilised to examine differences in psychological assessments between baseline and post-infusion measurements. Visualisation of results was conducted using BrainNet Viewer. The Pearson correlation coefficient was used to assess the association between psychological measurements and significant network metrics, with a significance level set at *α* = 0.05.

## Results

Table 1 presents the demographic and clinical characteristics of patients in the two clinical trials. Notably, patients in Trial 2 had a higher prevalence of previous suicide attempts compared with those in Trial 1 (*P* < 0.001). Both groups in each Trial demonstrated a significant reduction in MADRS scores and MADRS item 10 from baseline to Day 3 post-infusion (Supplementary Tables 1 and 2).

**Table 1:** Demographic and clinical characteristics of the two clinical trials

	Trial 1 (*n* = 45)	Trial 2 (*n* = 43)	*P*-value
Age (years, s.d.)	46.3 (10.92)	32.5 (11.49)	**<0.001**
Sex (*n*, %)			0.813
Female	32 (71.1)	32 (74.4)	
Male	13 (28.9)	11 (25.6)	
Age at disease onset (years, s.d.)	36.9 (11.38)	22.6 (7.30)	**<0.001**
Infusion group (*n*, %)			
0.5 mg/kg ketamine	14 (31.1)	21 (48.8)	
0.2 mg/kg ketamine	15 (33.3)	–	
Normal saline	16 (35.6)	–	
0.045 mg/kg midazolam	–	22 (51.2)	
History of attempted suicide (*n*, %)	18 (40.9)	40 (93.0)	**<0.001**
Total MADRS scores			
Baseline (s.d.)	32.82 (6.56)	37.42 (4.79)	**<0.001**
Day 3 post-infusion (s.d.)	23.00 (10.40)	28.86 (11.27)	**0.014**
MADRS item 10 scores			
Baseline (s.d.)	2.52 (1.30)	4.26 (0.44)	**<0.001**
Day 3 post-infusion (s.d.)	1.23 (1.12)	2.67 (1.52)	**<0.001**
Medications (*n*, %)			
Any antidepressant	44 (100.0)	39 (92.9)	0.112
Combined antidepressants	22 (50.0)	39 (92.9)	**<0.001**
Mood stabilisers	8 (18.0)	7 (16.7)	>0.999
Atypical antipsychotics	26 (59.1)	15 (35.7)	**0.034**
Benzodiazepines	37 (84.1)	37 (88.1)	0.758

MADRS, Montgomery–Åsberg Depression Rating Scale. Bold type indicates a statistical significance, *P* < 0.05.

### Graph theory findings of Trial 1

**Fig. 1:**
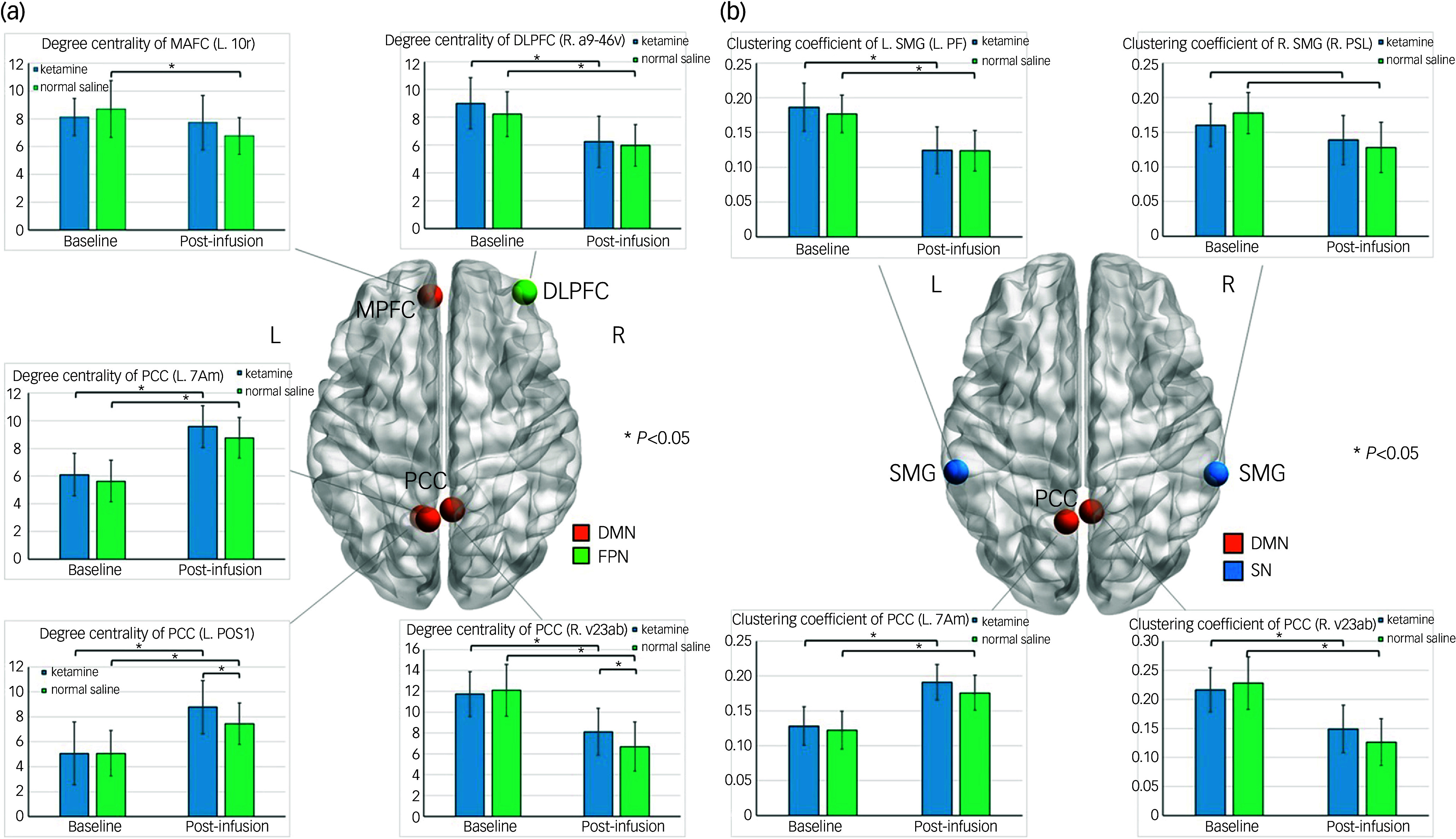
(a) Comparisons of degree centrality between infusion groups in clinical trial 1; (b) comparisons of clustering coefficient between infusion groups in Trial 1. DMN, default mode network; FPN, frontoparietal network; L, left; R, right; MPFC, medial prefrontal cortex; PCC, posterior cingulate cortex; DLPFC, dorsolateral prefrontal cortex; 10r, area 10r; 7Am, medial area 7A; POS1, parieto-occipital sulcus area 1; v23ab, area ventral 23 a + b; a9-46v, area anterior 9-46v; PF, area PF complex; PSL, perisylvian language area; SMG, supramarginal gyrus; SN, salience.

As detailed in Table 2 and Fig. 1, significant differences in degree centrality were observed within the DMN and FPN, particularly in the PCC, MPFC and DLPFC. The ANCOVA results revealed significant group-by-time interaction effects on the PCC and MPFC subregions (*P* = 0.033 and 0.025, respectively), as well as significant time effects on two PCC subregions and one DLPFC subregion (*P* = 0.019, 0.036 and 0.039, respectively). Regarding the significant group-by-time interaction effects, both the ketamine and normal saline group showed a decrease in degree centrality from Day 1 to Day 3 in the right PCC subregion, specifically in area ventral 23 a and b (v23ab). However, at Day 3 post-infusion, the ketamine group showed higher degree centrality in PCC v23ab than the normal saline group. Additionally, only the normal saline group evidenced a decrease in degree centrality from Day 1 to Day 3 in the MPFC area 10r subregion. Pertaining to the significant time effects, post hoc comparisons indicated that both the ketamine and normal saline group exhibited an increase in degree centrality from baseline to Day 3 post-infusion in the two left PCC subregions, namely medial area 7A (7Am) and parieto-occipital sulcus area 1 (POS1), while both groups showed a decrease in degree centrality from baseline to Day 3 in the right DLPFC subregion, specifically in area anterior 9-46v (a9-46v). Furthermore, the ketamine group evidenced higher degree centrality in the left PCC POS1 than the normal saline group at Day 3 post-infusion (Fig. 1a).

**Table 2:** Network metrics between infusion groups in Trials 1 and 2. This table presents the network metrics for brain regions, along with their corresponding Human Connectome Project extended atlas regions (shown in parentheses), that exhibited significant differences between the ketamine and normal saline groups in Trial 1 and between the ketamine and midazolam groups in Trial 2

Trial 1	Ketamine group (*n* = 29)	Normal saline group (*n* = 16)	Group effect (G)	Time effect (T)	G × T interaction	Post hoc comparison
**Degree centrality**						
MPFC (L. 10r)						
Baseline (s.d.)	8.1 (1.33)	8.7 (2.04)	0.668	0.524	**0.025**	Normal saline Day 3 < normal saline baseline
Day 3 post-infusion (s.d.)	7.7 (1.94)	6.8 (1.33)				
PCC (L. 7Am)						
Baseline (s.d.)	6.1 (1.53)	5.6 (1.51)	0.106	**0.036**	0.601	Ketamine Day 3 > ketamine baseline Normal saline Day3 > normal saline baseline
Day 3 post-infusion (s.d.)	9.6 (1.52)	8.8 (1.46)				
PCC (L. POS1)						
Baseline (s.d.)	5.1 (2.53)	5.1 (1.82)	0.284	**0.019**	0.086	Ketamine Day 3 > ketamine baseline Normal saline Day 3 > normal saline baseline Ketamine Day 3 > Normal saline Day 3
Day 3 post-infusion (s.d.)	8.8 (2.14)	7.4 (1.66)				
PCC (R. v23ab)						
Baseline (s.d.)	11.7 (2.16)	12.1 (2.50)	0.407	0.411	**0.033**	Ketamine Day 3 < ketamine baseline Normal saline Day3 < normal saline baseline Ketamine Day 3 > normal saline Day 3
Day 3 post-infusion (s.d.)	8.1 (2.25)	6.7 (2.36)				
DLPFC (R. a9-46v)						
Baseline (s.d.)	9.0 (1.85)	8.2 (1.62)	0.310	**0.039**	0.370	Ketamine Day 3 < ketamine baseline Normal saline Day 3 < normal saline baseline
Day 3 post-infusion (s.d.)	6.2 (1.84)	6.0 (1.50)				
**Clustering coefficient**						
PCC (L. 7Am)						
Baseline (s.d.)	0.13 (0.03)	0.12 (0.03)	0.118	**0.025**	0.409	Ketamine Day 3 > ketamine baseline Normal saline Day 3 > normal saline baseline
Day 3 post-infusion (s.d.)	0.19 (0.03)	0.18 (0.03)				
PCC (R. v23ab)						
Baseline (s.d.)	0.22 (0.04)	0.23 (2.50)	0.629	0.390	**0.019**	Ketamine Day 3 < ketamine baseline Normal saline Day 3 < normal saline baseline
Day 3 post-infusion (s.d.)	0.15 (0.04)	0.13 (0.04)				
L. SMG (L. PF)						
Baseline (s.d.)	0.17 (0.04)	0.18 (0.03)	0.532	**0.049**	0.491	Ketamine Day 3 < ketamine baseline Normal saline Day 3 < normal saline baseline
Day 3 post-infusion (s.d.)	0.13 (0.03)	0.12 (0.03)				
R. SMG (R. PSL)						
Baseline (s.d.)	0.16 (0.03)	0.18 (0.03)	0.716	0.854	**0.046**	Ketamine Day 3 < ketamine baseline; Normal saline Day 3 < normal saline baseline
Day 3 post-infusion (s.d.)	0.14 (0.04)	0.13 (0.04)				
**Characteristic path length**						
DLPFC (R. a9-46v)						
Baseline (s.d.)	3.73 (0.82)	4.01 (0.70)	0.497	**0.010**	0.371	Ketamine Day 3 > ketamine baseline Normal saline Day 3 > normal saline baseline
Day 3 post-infusion (s.d.)	5.45 (0.85)	5.49 (0.68)				

Trial 2	Ketamine group (*n* = 21)	Midazolam group (*n* = 22)	Group effect (G)	Time effect (T)	G × T interaction	Post hoc comparison
**Characteristic path length**						
PCC (L. 7Am)						
Baseline (s.d.)	4.18 (0.56)	3.98 (0.50)	0.893	0.370	**0.040**	Midazolam Day 3 > midazolam baseline
Day 3 post-infusion (s.d.)	4.14 (0.52)	4.31 (0.43)				

L, left; R, right; MPFC, medial prefrontal cortex; PCC, posterior cingulate cortex; DLPFC, dorsolateral prefrontal cortex; SMG, supramarginal gyrus; 10r, area 10r; 7Am, medial area 7A; POS1, parieto-occipital sulcus area 1; v23ab, area ventral 23 a + b; a9-46v, area anterior 9-46v; PF, area PF complex; PPC, posterior parietal cortex; PSL, perisylvian language area. Bold type indicates a statistical significance, *P* < 0.05.

Table 2 and Fig. 1b illustrate significant differences in clustering coefficient within the DMN and salience, particularly in bilateral PCC and SMG. The ANCOVA results revealed significant group-by-time interaction effects in the right PCC and SMG subregions (*P* = 0.019 and 0.046, respectively), as well as significant time effects in the left PCC and SMG subregions (*P* = 0.025 and 0.049, respectively). Regarding the significant group-by-time interaction effects, post hoc comparisons indicated that both the ketamine and normal saline group had a decrease in clustering coefficient from baseline to Day 3 post-infusion in the right PCC v23ab and right SMG perisylvian language area (PSL) subregions. Although there were no significant differences in Day 3 post-infusion clustering coefficient values between the two infusion groups in the right PCC v23ab and right SMG PSL subregions, the decreasing levels of clustering coefficient in those two subregions were lower in the ketamine group than in the normal saline group (Fig. 1b). Concerning the significant time effects, post hoc comparisons indicated that the ketamine and normal saline groups both showed an increase in clustering coefficient from baseline to Day 3 post-infusion in the left PCC 7Am subregion, and exhibited a decrease in clustering coefficient from baseline to Day 3 post-infusion in the left SMG area prefrontal complex subregion (Fig. 1b).

**Fig. 2:**
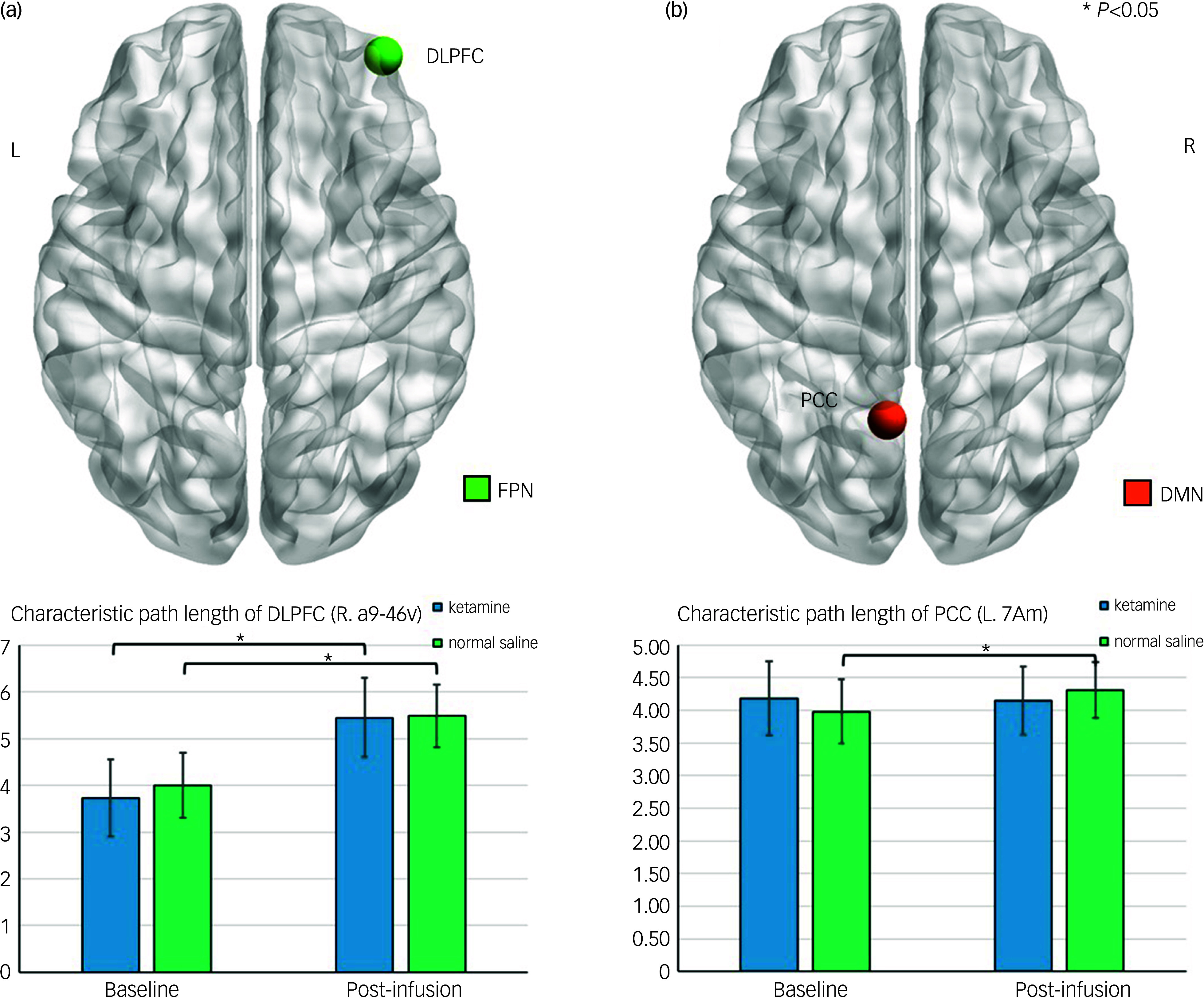
Comparisons of characteristic path length between infusion groups in (a) Trial 1 and (b) Trial 2. DMN, default mode network; FPN, frontoparietal network; L, left; R, right; DLPFC, dorsolateral prefrontal cortex; PCC, posterior cingulate cortex; 7Am, medial area 7A; a9-46v, area anterior 9-46v.

Table 2 and Fig. 2 further show a significant ANCOVA time effect on characteristic path length (*P* = 0.01), with both the ketamine and normal saline group showing an increase in characteristic path length from baseline to Day 3 post-infusion in the DLPFC a9-46v subregion.

### Graph theory findings of Trial 2

The ANCOVA results for graph theory metrics demonstrated a significant group-by-time interaction effect in the left PCC 7Am subregion (*P* = 0.040) in Trial 2 (Table 2 and Fig. 2). Specifically, at baseline, the ketamine group demonstrated a characteristic path length of 4.18 ± 0.56 while the midazolam group had a slightly lower value of 3.98 ± 0.50. At Day 3 post-infusion, the characteristic path length for the ketamine group decreased slightly to 4.14 ± 0.52 whereas that for the midazolam group increased significantly to 4.31 ± 0.43.

Finally, in terms of transitivity and intra- and inter-network functional connectivity, neither clinical trial exhibited significant differences. Additionally, there were no significant correlations between network metrics and symptom assessments in either Trial 1 or 2.

## Discussion

To the best of our knowledge, the present study is the first graph theory-based functional connectivity analysis based on the HCPex atlas that elucidates the effect of ketamine on DMN, salience and FPN networks among patients with TRD.

A particularly noteworthy finding of our study is the observation that low-dose ketamine modulated brain activity in both task-positive networks, especially salience, as well as in the task-negative network (DMN), to exert antidepressant effects. The most significant ketamine-related changes in the graph theory metrics in our study were the reduced degree centrality and clustering coefficient in the right PCC v23ab (DMN), and the decreased clustering coefficient in the right SMG PSL (salience), particularly in Trial 1. As mentioned previously, changes within non-traditional hub regions in a network may contribute to subtle but important network reconfiguration.^10,11,16^ PCC v23ab plays a crucial role in an internally focused human state, including the retrieval of semantic and episodic memories.^17^ Overactivation of PCC v23ab impairs self-relevant functions, which may reflect the occurrence of rumination, guilt and suicidal thoughts among patients with depression.^17,18^ An rs–fMRI study of 31 patients with major depressive disorder and suicidal ideation (MD–SI), 56 patients with depression but without suicidal ideation (MD–nSI) and 48 healthy controls demonstrated that the MD–SI group exhibited increased functional connectivity variability between the left ventral PCC (PCC v23ab) and the left inferior frontal gyrus compared with the MD–nSI group.^18^ Cheng et al revealed that patients with major depressive disorder had significantly higher functional connectivity between the ventral PCC and lateral orbitofrontal cortex compared with healthy controls. They also found that regular treatment with antidepressants normalised this increased functional connectivity among patients.^19^ In Trial 1, we found that low-dose ketamine reduced the degree centrality and clustering coefficient in PCC v23ab among patients with TRD, as did the normal saline placebo. Interestingly, we further found that Day 3 post-infusion degree centrality was higher in the ketamine group than in the normal saline group. This counterintuitive finding may indicate two issues: first, a decrease in functional connectivity in the DMN was associated with an improvement in depressive symptoms; and second, ketamine’s antidepressant effect may be related to an optimal reduction in DMN function, which could not be achieved by the normal saline placebo. However, the present study failed to find associations between changes in graph theory metrics and those in mood and suicidal symptoms.

The SMG PSL is responsible not only for speech and language functions but also for various high-level cognitive functions, including essential information processing and meta-control of cognitive functions.^20^ Tsuchiyagaito et al assessed resting-state functional connectivity differences between 50 patients with major depressive disorder who exhibited high repetitive negative thinking (RNT) and 50 who exhibited low RNT.^21^ They discovered that the intensity of RNT was associated with increased functional connectivity between the SMG PSL and both the anterior insula and DLPFC,^21^ suggesting increased functional connectivity within salience and between that and the FPN, which are considered core pathomechanisms of TRD.^22,23^ Evidence has shown that patients with TRD who responded to various intensive treatment strategies, including repetitive transcranial magnetic stimulation, electroconvulsive therapy and low-dose ketamine infusion, experienced decreased salience functional connectivity in the post-treatment period.^22,23^ Vasavada et al reported that, in patients with TRD, serial infusions of low-dose ketamine resulted in decreased functional connectivity in salience. Additionally, the observed reduction in salience-related functional connectivity was associated with improved behavioural inhibition.^24^ Our findings showed that both low-dose ketamine and normal saline reduced the clustering coefficient of the right SMG PSL. However, these values decreased from 0.16 to 0.14 in the ketamine group and from 0.18 to 0.13 in the normal saline group, resulting in a significant group-by-time effect. This again indicates that low-dose ketamine may modulate salience – not too much and not too little. The antidepressant effect of low-dose ketamine may be associated with this optimal modulation.

Surprisingly, we found a group-by-time interaction effect on characteristic path length only in the left PCC 7Am (DMN) in Trial 2. This significant interaction effect was primarily driven by the greater increase in characteristic path length in the midazolam group. As previously mentioned, increased characteristic path length indicated a less integrated network,^15^ which may negatively impact the treatment of depression. The PCC 7Am plays an important role in cognitive and affective functions, including working memory, planning and performance monitoring, sensorimotor dynamics and interoception.^25^ Reed et al reported an emotion-mediated effect of low-dose ketamine on PCC 7Am activity, indicating that decreased depressive symptoms following a single infusion of 0.5 mg/kg ketamine were associated with reduced activation to angry-face trials and greater activation to happy-face trials during the emotional-face stimuli task.^25^ Despite no significant difference in ketamine-related changes in the graph theory metrics in Trial 2, the midazolam-related increase in characteristic path length in the left PCC 7Am may suggest the poor antidepressant effect of midazolam among patients with TRD.

In addition to the group-by-time interaction effect of low-dose ketamine and placebo infusions in patients with TRD, we also found a meaningful time effect of low-dose ketamine on degree centrality of the left PCC POS1 in Trial 1. Both low-dose ketamine and normal saline infusions increased degree centrality of the left PCC POS1, being significantly higher in the ketamine group than in the normal saline group at Day 3 post-infusion. PCC POS1 is responsible for episodic memory and navigation, scene representations reaching the hippocampal system and synergistic work with the DLPFC during planning.^26,27^ Taylor et al demonstrated that patients who were depressed exhibited higher glutamate levels in the parieto-occipital sulcus region compared with the control group,^27^ supporting the notion of glutamatergic system dysfunction in major depressive disorder. This finding may echo our finding that increased degree centrality in the left PCC POS1 may play a potential role in the antidepressant effect of ketamine.

The placebo effect may help us understand the other time effects on the graph theory metrics in Trial 1. These included increased degree centrality and clustering coefficient in the left PCC 7Am, decreased degree centrality in the right DLPFC a9-46v, decreased clustering coefficient in the left SMG PF and increased characteristic path length in the right DLPFC a9-46v following a single infusion of either ketamine or placebo. Evidence has shown an association between overactivation in the right DLPFC and TRD.^28^ Additionally, the increased degree centrality and clustering coefficient in the left PCC 7Am in both infusion groups in Trial 1, along with the increased characteristic path length in the left PCC 7Am only in the midazolam group in Trial 2, may suggest a potential association between the left PCC 7Am and antidepressant effect. An increase in degree centrality in the left DMN (PCC 7Am and POS1) and a reduction in the right DMN (PCC v23ab) may further support the hypothesis of disrupted hemispheric connectivity specialisation in the pathomechanism of depression.^28^

Finally, an unexpected but important issue was that the changes in ketamine-related, graph theory metrics were observed in Trial 1 but not in Trial 2. In the latter, the only significant finding of the group-by-time effect in characteristic path length in the left PCC 7Am was primarily contributed by the effect from midazolam, rather than from ketamine. Such a notable discrepancy between the findings in Trials 1 and 2 may echo our previous study on ketamine-related changes in corticothalamic functional connectivity, which reported 18 significant findings in Trial 1 and only four in Trial 2.^29^ Additionally, we found no associations between changes in graph-theory metrics and those in clinical symptoms in the present study. Further investigation is necessary to determine whether the effect of ketamine on the triple networks is the core neuromechanism underlying its antidepressant effect.

This study has several limitations. First, both clinical trials employed an add-on design, which allowed the participants to continue their existing medication regimen. Although this approach prioritised patient safety, particularly for those with severe suicidal symptoms, it may have influenced study outcomes. Second, the use of various placebos (normal saline and midazolam) in the two trials precluded direct head-to-head comparisons. Third, the heterogeneity in demographic and clinical characteristics between study populations may have limited cross-trial comparisons. Finally, the administration of a single infusion of low-dose ketamine in both trials necessitates further investigation to determine the generalisability of our findings to repeated ketamine infusion regimens.

In conclusion, low-dose ketamine altered the functional connectivity-based, graph-theory metrics, including reduced degree centrality and clustering coefficient in the right PCC v23ab (DMN), reduced clustering coefficient in the right SMG PSL (salience) and increased characteristic path length in the right DLPFC a9-46v (FPN) among patients with TRD. These results underscore the potential utility of the triple network model in elucidating the mechanisms underlying the antidepressant effect of ketamine.

## Supporting information

Lin et al. supplementary material Lin et al. supplementary material

## Data Availability

The data that support the findings of this study are available from the corresponding author, M.-H.C., on reasonable request, owing to the ethical regulation of the institutional review boards of Taipei Veterans General Hospital.

## Supplementary material

The supplementary material for this article can be found at https://doi.org/10.1192/bjp.2025.14
