# Supplementary material for: Triple-network model–based graph theory analysis of the effectiveness of low-dose ketamine in patients with treatment-resistant depression: two resting-state functional MRI clinical trials
Source: Br J Psychiatry. 2025 Apr 2;227(5):766–74. doi: 10.1192/bjp.2025.14 (PMC12550656; doi:10.1192/bjp.2025.14)
Supplement: Lin et al. supplementary material [file S0007125025000145sup001.docx]

Supplementary figure 1. Flowchart of clinical trial 1

We excluded the MRI data of three participants due to the imaging quality. So, finally, 45 participants were analyzed for the functional connectivity.

Supplementary figure 1. Flowchart of clinical trial 2.


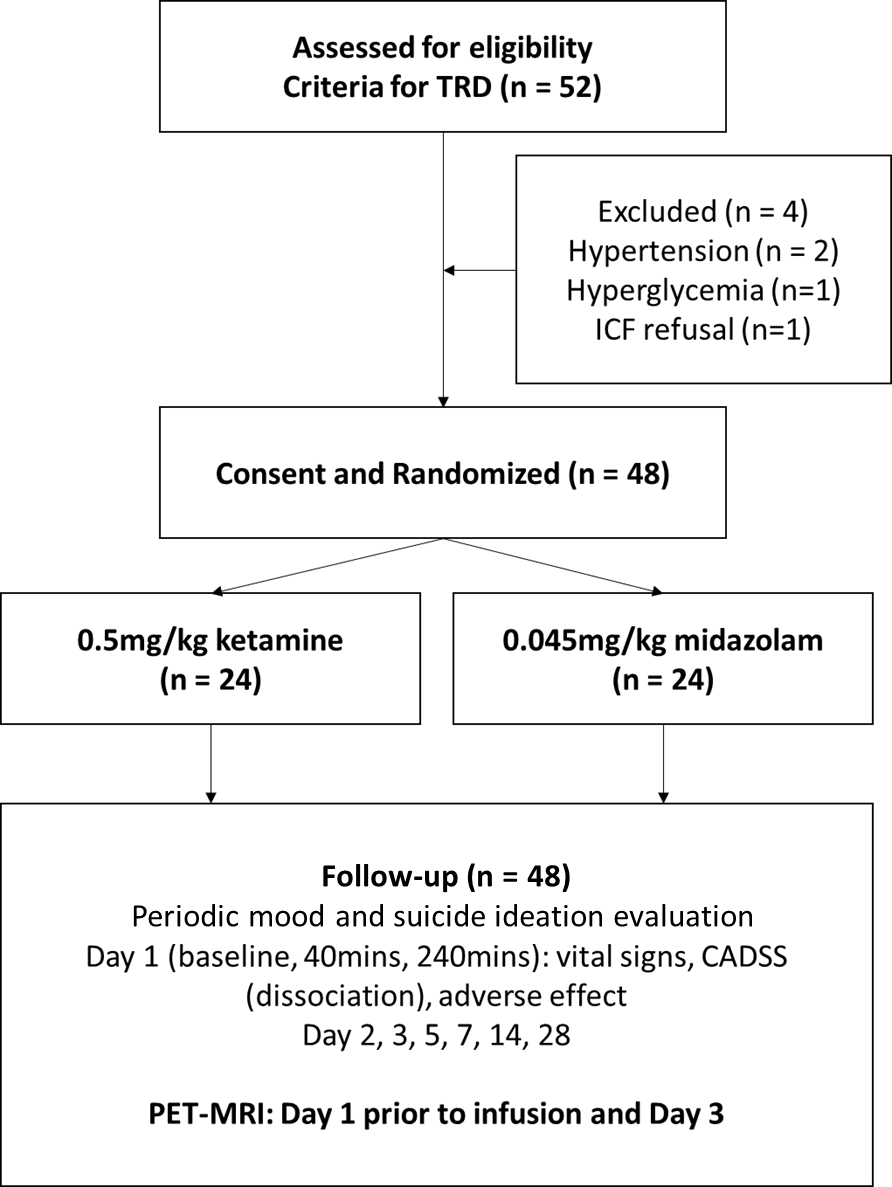


We excluded the MRI data of five participants due to the imaging quality. So, finally, 43 participants were analyzed for the functional connectivity.

Supplementary Table1. Summary of clinical score differences in clinical trial 1

|  | Baseline | Day 3 | Difference | p-value (Within Group) | Baseline  p-value (Between Groups) | Day 3  p-value (Between Groups) |
| --- | --- | --- | --- | --- | --- | --- |
| Ketamine group (n = 29) |  |  |  |  |  |  |
| Total MADRS scores (SD) | 32.14 (7.41) | 21.25  (11.32) | 10.9  (9.13) | **<0.001** | 0.316 | 0.142 |
| MADRS item 10 scores (SD) | 2.46  (1.32) | 0.96  (0.88) | 1.5  (1.32) | **<0.001** | 0.699 | 0.068 |
| Normal saline group (n = 16) |  |  |  |  |  |  |
| Total MADRS scores (SD) | 34.00  (4.70) | 26.06  (7.97) | 7.93  (6.35) | **<0.001** |  |  |
| MADRS item 10 scores (SD) | 2.63  (1.30) | 1.69  (1.35) | 0.94  (1.12) | **0.004** |  |  |

SD: standard deviation; MADRS: Montgomery–Åsberg Depression Rating Scale.

Supplementary Table 2. Summary of clinical score differences in clinical trial 2

|  | Baseline | Day 3 | Difference | p-value | Baseline  p-value (Between Groups) | Day 3  p-value (Between Groups) |
| --- | --- | --- | --- | --- | --- | --- |
| Ketamine group (n =21) |  |  |  |  |  |  |
| Total MADRS scores (SD) | 35.62  (4.85) | 23.52  (11.36) | 12.10  (10.47) | **<0.001** | **0.014** | **0.002** |
| MADRS item 10 scores (SD) | 4.14  (0.36) | 2.10  (1.45) | 2.05  (1.36) | **<0.001** | 0.100 | **0.013** |
| Midazolam group (n =22) |  |  |  |  |  |  |
| Total MADRS scores (SD) | 39.14  (4.14) | 33.95  (8.68) | 5.18  (7.63) | **0.004** |  |  |
| MADRS item 10 scores (SD) | 4.36  (0.49) | 3.23  (1.42) | 1.14  (1.36) | **0.001** |  |  |

SD: standard deviation; MADRS: Montgomery–Åsberg Depression Rating Scale.
